# Role of the DPP4 Receptor in SARS‐CoV Entry: Insights From Docking and Molecular Dynamics Simulations

**DOI:** 10.1002/prot.70011

**Published:** 2025-07-02

**Authors:** Patrícia Pereira Duzi Carvalho, Nelson Augusto Alves

**Affiliations:** ^1^ Department of Physics, FFCLRP University of São Paulo Ribeirão Preto Brazil

**Keywords:** ACE2, dissociation energy, DPP4, free energy, MERS‐CoV, molecular dynamics, SARS‐CoV, SMOG

## Abstract

Protein–receptor interactions play a critical role in viral entry and pathogenesis. While ACE2 is the primary receptor for SARS‐CoV, the role of DPP4 as potential coreceptor remains underexplored. This study investigates the binding mechanisms and dissociation dynamics of the SARS‐CoV/DPP4, SARS‐CoV/ACE2 and MERS‐CoV/DPP4 complexes using molecular docking and molecular dynamics simulations. The SARS‐CoV/DPP4 complex exhibited the highest free‐energy barrier ($\Delta F=6.77\;k_{B}T$), suggesting significant stability despite being energetically unfavorable. In contrast, the MERS‐CoV/DPP4 complex, with the lowest free‐energy barrier ($\Delta F=2.17\;k_{B}T$), was the most likely to form and the least resistant to dissociation. The SARS‐CoV/ACE2 complex demonstrated the highest $Q_{\text{bound}}$, reflecting well‐organized interfacial side chains that facilitate hydrogen bonding, yet its relatively low free‐energy barrier and dissociation temperature made it prone to dissociation. These findings highlight an inverse relationship between electrostatic complementarity and protein–protein complex stability, where increased electrostatic complementarity correlates with reduced stability due to frustration from competing interactions. While DPP4 may serve as a coreceptor for SARS‐CoV, its interaction is constrained by significant energy barriers, suggesting it may only occur under specific biological conditions or alternative binding pathways.

## 1 Introduction

Coronaviruses (CoVs) belong to a family of viruses known as Coronaviridae. Within the subfamily Orthocoronavirinae, four genera—α, β, γ, and δ‐coronavirus—have been identified based on phylogenetic analysis. These viruses exhibit a wide range of infectivity across mammalian and avian species. The α and β genera primarily infect mammals, including humans, while the γ and δ genera predominantly affect mammals and birds. Notably, three CoVs within the β genus have gained significant attention due to their ability to cause severe respiratory diseases: SARS‐CoV (severe acute respiratory syndrome coronavirus), SARS‐CoV‐2, and MERS‐CoV (Middle East respiratory syndrome coronavirus). Among these, SARS‐CoV‐2 stands out for its heightened lethality. SARS‐CoV and MERS‐CoV are also considered highly pathogenic and are known to be transmitted from bats to humans via intermediate hosts, specifically palm civets and dromedary camels [1].

Several CoVs utilize angiotensin‐converting enzyme 2 (ACE2) as the cell surface receptor to enter human cells via endocytosis. Examples include the above‐mentioned β‐genus human SARS‐CoV [2, 3], and SARS‐CoV‐2 [4, 5, 6, 7].

The successful initial interaction between the virus and host cells is fundamental for initiating the reproductive viral cycle and establishing an effective infection. The recognition of the molecular receptor is initiated by the spike protein located on the virion envelope, primarily through the receptor‐binding domain (RBD). All spike proteins of SARS‐CoV, SARS‐CoV‐2, and MERS‐CoV are homotrimeric glycoproteins, a structural hallmark of coronaviruses. Each monomer of the spike protein consists of two main subunits, S1 and S2. The S1 subunit contains the RBD region, while the S2 subunit facilitates the membrane fusion between the virus and the host cell.

The infectivity of SARS‐CoV strains varies widely, possibly linked to their binding affinities to ACE2, which may correlate with the severity of the disease in humans [8]. In contrast, the MERS‐CoV and its genetically related bat coronavirus have developed a remarkable ability to utilize dipeptidyl peptidase 4 (DPP4), a serine protease, as their viral receptor [9, 10, 11]. While this specificity in receptor usage among coronavirus species suggests distinct mechanisms of cell entry, it does not exclude the possibility of coreceptor involvement. As a matter of fact, viruses have developed diverse genome replication and protein expression strategies to exploit the cell host translational machinery over time [12, 13, 14].

It is known that proteases in the lung are the primary sites for coronavirus infection, despite their low expression of ACE2. This suggests that both membrane proteins ACE2 and DPP4 are relevant for the severity of infections in the lungs, as well as in other tissues [15, 16]. Thus, the utilization of DPP4 by SARS‐CoV and SARS‐CoV‐2 may result not only from its binding preference but also from proteases abundantly distributed in human tissues [17].

The investigation into the potential use of the DPP4 receptor by both SARS‐CoV‐2 and SARS‐CoV has attracted significant attention in understanding possible alternative mechanisms underlying viral entry into the host cell. Despite diverse investigations employing techniques such as experimental assays, molecular docking, and molecular dynamics (MD) simulations, the results do not support significant utilization of DPP4 as an alternate route for viral cell entry by either SARS‐CoV‐2 [5, 17, 18, 19, 20] or SARS‐CoV [17]. On the other hand, clinical data obtained with DPP4 inhibitors indicate that they are prominent pharmacological approaches against coronavirus infections, leading us to conclude that they work to inhibit viral replication [15, 16, 21, 22, 23]. Therefore, while the current findings may suggest limited involvement of DPP4 as an alternative receptor for SARS‐CoV, clinical results present compelling reasons to investigate the competition between the host receptors ACE2 and DPP4 by the coronavirus, as well as the apparent preference for ACE2.

The entry of the coronavirus is a multi‐stage process that involves receptor engagement, protease processing, and membrane fusion. Exposing the fusion peptide located in the S2 subunit is a critical stage in initiating the membrane fusion process because it is essential for the viral infection. After the fusion peptide is inserted into the host cell membrane, the spike protein must dissociate from its receptor to reach its postfusion state [4, 6, 24]. A strong interaction could impede this dissociation, thereby interfering with successful membrane fusion. Thus, our approach focuses on the dissociation dynamics of protein–receptor complexes, going beyond the usual binding energy calculations [19, 25].

As a matter of fact, our approach enables us to investigate the hypothesis that, although SARS‐CoV may exhibit stronger binding affinity for ACE2, the widespread distribution of DPP4 could allow it to function effectively as an alternative receptor, provided that the free‐energy barrier of dissociation for the SARS‐CoV/DPP4 complex yields comparable values. A small free‐energy barrier of dissociation from the receptor indicates a weak interaction. Such a weak interaction is necessary to allow conformational changes in the spike protein, which are crucial for exposing the fusion peptide and enabling the membrane fusion. Conversely, a large free‐energy barrier may hinder, delay, or even prevent the fusion process.

To test our hypothesis, molecular docking simulations were initially conducted to model the interaction between the RBD region of the SARS‐CoV spike protein and the human DPP4 receptor. Following this, MD simulations were performed across a range of temperatures to investigate the dissociation of the protein complex over time. All simulations utilized the proposed molecular complex model as the initial conformation. Furthermore, MD simulations were carried out for the SARS‐CoV/ACE2 and MERS‐CoV/DPP4 molecular complexes using the same force field, enabling a comparative analysis of the estimated free‐energy barriers.

MD simulations of molecular complexes performed across a range of temperatures can identify the temperatures at which the total energy exceeds the Helmholtz free‐energy barrier $\Delta F_{\text{dissoc}}$. Once dissociation occurs, it is unlikely for the system to return to the bound state due to the vast conformational space available to the unbound molecules under these environmental conditions. The free‐energy change $\Delta F_{\text{dissoc}}$ is related to the dissociation constant $K_{d}$,

$$\Delta F_{\text{dissoc}}=k_{B}T\ln K_{d}.$$

This quantity differs from the binding free‐energy $\Delta F_{\text{bind}}$, which represents the energy required to associate protein and receptor to form a stable complex. The binding free‐energy $\Delta F_{\text{bind}}$ is defined as

$$\Delta F_{\text{bind}}=-k_{B}T\ln K_{a},$$

where $K_{a}$ is the association constant. Under ideal thermodynamic conditions, $K_{d}=1/K_{a}$. However, in nonideal systems where allosteric effects and intermediate states are present, $K_{d}\neq 1/K_{a}$. Thus, it is essential to directly estimate $\Delta F_{\text{dissoc}}$ using numerical methods. For simplicity, we will denote this numerically estimated free energy of dissociation as ΔF.

## 2 Methods

### 2.1 Active Binding Sites of the Molecular Complexes

Before proceeding with the docking formation between SARS‐CoV and DPP4, it is essential to identify the active binding sites that establish the SARS‐CoV/ACE2 and MERS‐CoV/DPP4 molecular complexes. These binding sites serve as the foundation for analyzing the docking results and selecting appropriate molecular models for subsequent computational simulations.

**FIGURE 1:**
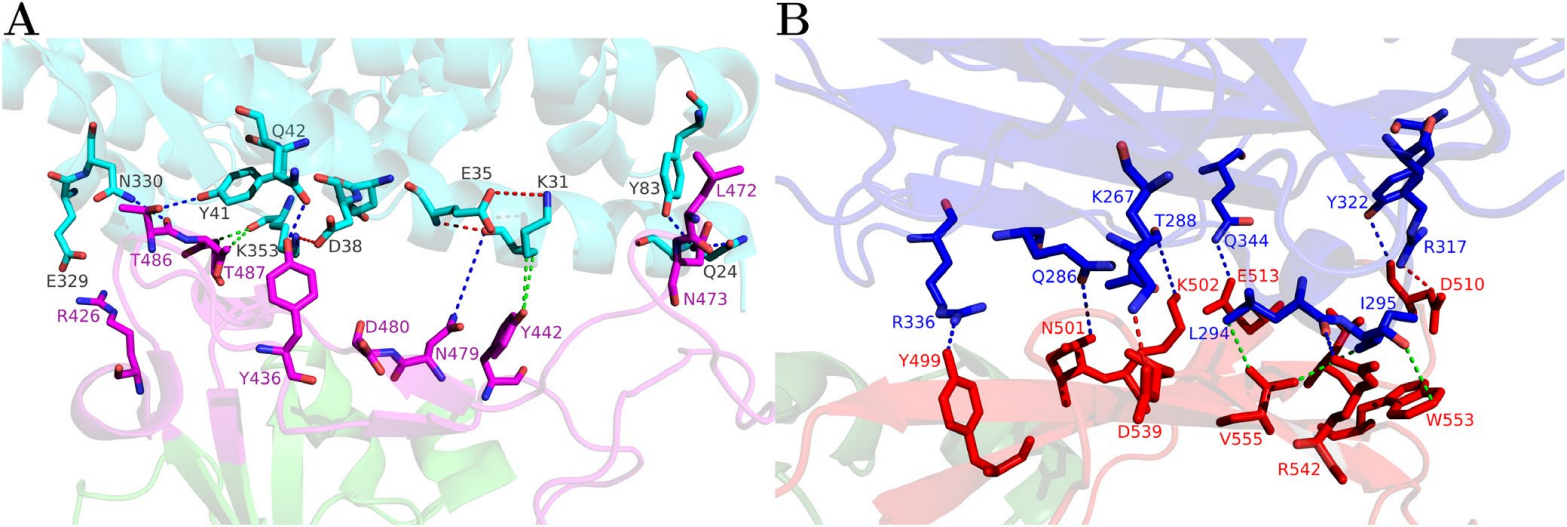
(A) Interaction surface between SARS‐CoV and ACE2 receptor. The RBM, comprising residues N424‐Y494 of SARS‐CoV, is highlighted in magenta, while the ACE2 receptor is depicted in cyan. (B) Interaction surface between MERS‐CoV and DPP4 receptor. The RBM, comprising residues 484–567 of MERS‐CoV, is highlighted in red, while the DPP4 receptor is depicted in blue.

The spike RBD of SARS‐CoV is the only domain that contacts ACE2 directly, and it is constituted by the residues R306‐F527. While RBD in spike proteins is determinant for the virus‐receptor interaction, the active residues lie in the region termed the receptor‐binding motif (RBM), which is part of RBD and plays a fundamental role in binding to the outer surface of their receptors. The RBM region comprises the residues N424‐Y494 and forms the hydrophobic pocket described in [26]. Due to the presence of this hydrophobic pocket, the amino acid residues responsible for the binding interactions are situated in close proximity to this conformational structure (Figure 1A). For instance, residues N479 and T487 have been identified as essential for SARS‐CoV spike RBD/ACE2 binding [27, 28]. The residue N479 forms hydrogen bonds (H‐bonds) with residues K31 and H34 of ACE2. Residue K31 forms a salt bridge with E35, both of which are buried within that hydrophobic environment. Similarly, residue T487 forms H‐bonds with Y41 of ACE2. Residue Y41 is positioned near K353 on ACE2, forming a salt bridge with D38, all also buried in the pocket. Other significant residues contributing to this attachment through H‐bond contacts with ACE2 include Y475 and T486.

The MERS‐CoV RBD region consists of residues E367 to Y606 and can easily adjust to the host through its four external β‐strands, which create a relatively flat surface for interaction with the two‐bladed β‐propeller domain and the α/β hydrolase domain of DPP4 (Figure 1B).

The DPP4 receptor features an extracellular domain composed of 728 residues and exhibits a binding interface characterized by a group of hydrophilic residues that form specific polar contacts: hydrogen bonds and salt bridge contacts [9, 10, 17, 29, 30, 31]. Among the MERS‐CoV RBD residues that directly interact with DPP4 through H‐bonds are RBD Y499 with DPP4 R336, RBD N501 with DPP4 Q286, RBD K502 with DPP4 T288, and RBD E513 with DPP4 Q344. A short α‐helix of DPP4 located at the interface forms a hydrophobic core consisting of residues A291, L294, and I295 that are in close proximity to MERS‐CoV RBD residues Y540, W553, and V555. Furthermore, salt bridges are formed, specifically through interactions between RBD D510 and DPP4 R317, as well as between RBD D539 and DPP4 K267.

### 2.2 Structure Prediction—Homology Modeling

Homology modeling was employed to investigate the spike/receptor interaction by predicting the missing residues in the structure of the spike protein (PDB ID:2AJF), which was resolved at 2.90 Å through x‐ray diffraction. Specifically, six residues (Asp 376, Leu 377, Cys 378, Phe 379, Ser 380, and Asn 381) located in the RBD of the SARS‐CoV spike protein were modeled. This was achieved using MODELLER 10.1 [32] along with Python scripts to facilitate the modeling process.

### 2.3 Data Acquisition and Molecular Docking Simulations

The conformational structure of SARS‐CoV complexed with the DPP4 receptor was generated through specialized molecular docking simulations using the computational tool HADDOCK 2.4 [33]. Among the evaluated docking softwares, HADDOCK has exhibited superior performance in generating high‐quality poses and accurately predicting protein–protein interactions. Its ability to incorporate experimental data to guide the docking process has resulted in top‐ranking classification among all participants in the CASP‐CAPRI Round 1 [34]. To optimize our molecular docking of the SARS‐CoV/DPP4 complex, we utilized the complete crystallographic structure of the DPP4 conformation to achieve optimal results from HADDOCK. This approach involved using residues S39 to P766 (PDB ID:1NU6) as a template, with additional details provided in Supporting Information.

**FIGURE 2:**
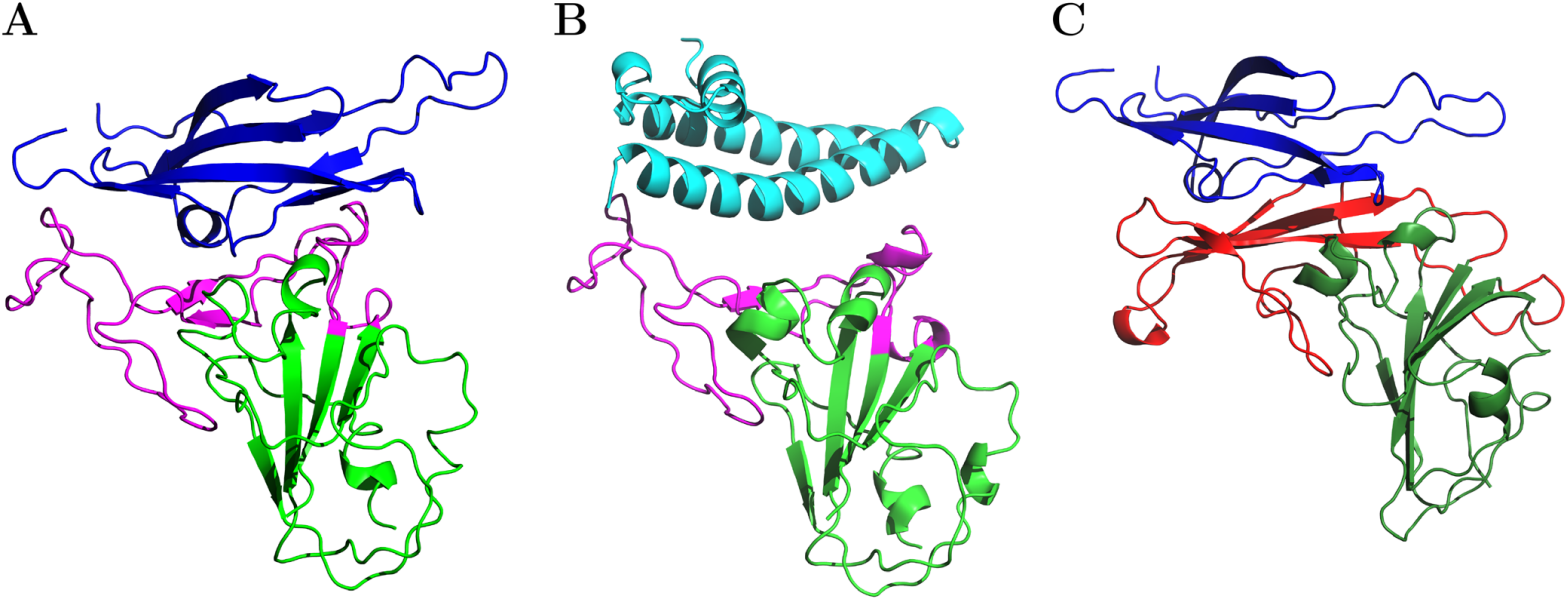
Molecular models used to perform molecular dynamics simulations. (A) SARS‐CoV complexed with human DPP4 receptor. The RBD (residues 323–502) is depicted in magenta and green. The magenta color highlights its RBM region. The selected DPP4 residues (residues 259–353) are shown in blue. (B) SARS‐CoV complexed with human ACE2 receptor. The RBD is depicted in magenta (RBM) and green. The ACE2 (residues 19–103) receptor is shown in cyan. (C) MERS‐CoV complexed with human DPP4 receptor. The RBD (residues 382–585) is depicted in red (RBM) and green. The DPP4 receptor is shown in blue.

Given that the specific molecular recognition of host receptors is mediated by the RBDs, we focused our MD simulations on the SARS‐CoV/DPP4 complex, restricting the SARS‐CoV modeling to residues C323–E502. This selection encompasses almost the entire RBD of SARS‐CoV and includes all essential residues involved in receptor recognition. To complete our modeling, we restricted the DPP4 receptor to residues A259 to W353, as this range includes all key residues critical for the interaction. Consequently, the final modeling for the MD simulations is depicted in Figure 2A.

Additionally, we also modeled SARS‐CoV/ACE2 and MERS‐CoV/DPP4 complexes using the defined specific residue ranges from the SARS‐CoV/DPP4 modeling to conduct a comparative MD analysis. For the SARS‐CoV/ACE2 complex (PDB ID:2AJF), we kept the selected residue range 323–502 of the SARS‐CoV RBD and residues S19 to N103 of the ACE2 receptor (Figure 2B). In the case of the MERS‐CoV/DPP4 complex (PDB ID: 4L72), we selected residues E382 to C585 of the MERS‐CoV RBD and the previously selected residues 259–353 of the DPP4 receptor (Figure 2C).

### 2.4 Structure Validation

The structural evaluation tool available on the MolProbity web server (http://molprobity.biochem.duke.edu) [35] was employed to assess the quality of the constructed models by analyzing their stereochemical properties. Additionally, the RMSD was calculated by aligning the modeled protein with the crystallographic structure of the SARS‐CoV RBD. The validation of the SARS‐CoV/DPP4 modeling is presented in Figures S1A and S2.

### 2.5 Molecular Dynamics Simulation

MD simulations were performed using GROMACS 5.1.4 to obtain the thermodynamic properties of the SARS‐CoV/DPP4, SARS‐CoV/ACE2, and MERS‐CoV/DPP4 complexes. These simulations incorporated a potential energy function based on the energy landscape theory [36]. This approach employs a structure‐based model (SBM) with a Hamiltonian whose global minimum is defined by the input molecular structure, thus imposing an explicit native bias toward the conformational structure of the proteins [37]. The input data for modeling was prepared using SMOG (Structure‐based MOdels in GROMACS) [38]. This preparation focused solely on heavy atoms, with each atom being represented by unit mass.

Harmonic potentials were employed to maintain bond lengths, bond angles, improper dihedrals, and planar dihedrals. Unbound atom pairs in contact in their native state were modeled using the Lennard–Jones potential, while other nonlocal interactions were kept repulsive. The bond, angle, improper, and planar terms were used to maintain the backbone's geometry, with flexible dihedrals modeled by cosine functions. The full set of SBM atomic parameters was used to describe interatomic native contacts between the proteins. A cutoff distance of 4 Å was applied to all short‐range interactions through the introduction of the Shadow contact map defined in the SMOG software.

All three systems were centered in a cubic box applying periodic boundary conditions, with an edge length of 90 Å. The equilibration phase lasted 3 ns, followed by 200 ns of MD simulations performed under isochoric‐isothermal conditions (NVT ensemble), with a time step of 0.002 ps. Preliminary tests indicated that 200 ns were sufficient to observe the dissociation dynamics of the three molecular complexes. For each complex, simulations were performed at 32 different temperatures (expressed in reduced units). These temperature sets were selected based on preliminary tests to ensure that the histograms for energy and the reaction coordinate, fraction of native intermolecular contacts, would exhibit overlaps across the successive temperatures.

The total energy (*E*), root‐mean‐square deviation (RMSD), and radius of gyration (*R*_gyr_) were calculated using the analysis tools contained within the GROMACS software package. The fraction of native intermolecular contacts (Q) was determined based on the following definition of a native contact: a contact is considered a native contact if the residue pair has atoms within 1.5 of their native distances. The analysis of the free energy of dissociation for each complexation was conducted using the weighted histogram analysis method (WHAM) [39]. This approach enabled us to combine data from simulations performed at various temperatures into an unique free‐energy profile. Here, we employed the Pywham script (available at https://sourceforge.net/projects/pywham/) to facilitate this process.

## 3 Results

### 3.1 Binding Energies Predicted by HADDOCK Modeling

**FIGURE 3:**
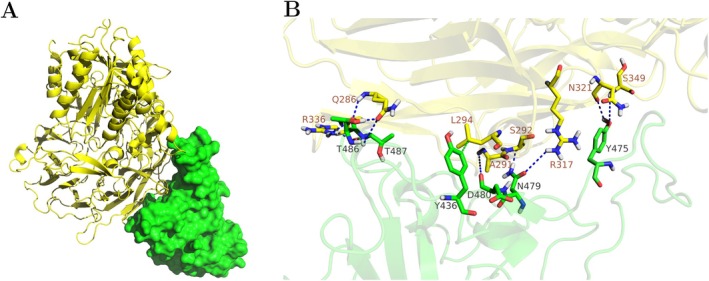
(A) The complete molecular complex of SARS‐CoV and DPP4, as predicted by HADDOCK. The SARS‐CoV RBD is depicted in green, while DPP4 is in yellow. The top‐ranked predicted complex retained the characteristic configurational pocket of the SARS‐CoV RBD, highlighting the shape complementarity between the binding interfaces. (B) The binding interface illustrating the interaction between the spike protein of SARS‐CoV and the DPP4 receptor of MERS‐CoV.

Figure 3A displays the top molecular docking prediction between SARS‐CoV and DPP4 utilizing the complete structure of DPP4. Notably, this modeling preserves the hydrophobic pocket in the SARS‐CoV RBM characterized by the hydrophobic sequence Y481‐G482‐F483‐Y484. The shape complementarity between the interacting molecular surfaces is crucial for recognition and binding affinity of SARS‐CoV to ACE2 [26]. The essential residues involved in the SARS‐CoV/ACE2 interaction continue to play a significant role in facilitating the binding of SARS‐CoV to the DPP4 receptor. Table S1 shows that the DPP4 residues interacting with the MERS‐CoV RBD interfacial residues also form hydrogen bonds with SARS‐CoV. Specifically, the SARS‐CoV RBM residue N479 forms H‐bond contacts with S292 and R317 in the DPP4 receptor, while residue T487 forms H‐bond contacts with Q286 and R336 (Figure 3B). Other SARS‐CoV RBM residues critical for SARS‐CoV/ACE2 binding, such as Y475 and T486, also form H‐bond contacts with DPP4 residues N321 and S349, and Q286 and R336, respectively. Some residues, like L472 and D480, contribute to repulsive interactions.

The crystal structures of SARS‐CoV/ACE2 and MERS‐CoV/DPP4 complexes were also submitted to HADDOCK 2.4 for comparative analysis, as summarized in Table 1. It is noteworthy that the binding energy of the SARS‐CoV/ACE2 complex, along with its energy components, closely aligns with the values reported for the SARS‐CoV‐2/ACE2 complex in [19]. The SARS‐CoV‐2/ACE2 complex exhibits [19] a more favorable binding energy compared to the SARS‐CoV/ACE2 complex, with a relative difference of 9%, reinforcing the well‐documented favorable interaction between SARS‐CoV‐2 and its ACE2 receptor.

**TABLE 1:** Binding interaction energies (kcal/mol) for the MERS‐CoV/DPP4, SARS‐CoV/ACE2, and SARS‐CoV/DPP4 complexes, estimated using the computational tool HADDOCK.

Haddock summary	MERS‐CoV/DPP4	SARS‐CoV/ACE2	SARS‐CoV/DPP4
van der Waals	−90.62	−77.53	−103.68
Electrostatic	−453.03	−219.46	−157.05
Desolvation	2.40	−19.75	−18.78
Buried surface area	3093.41	2316.19	2741.13
Binding energy	−368.15	−240.39	−155.03

The energy components in Table 1 show that the MERS‐CoV/DPP4 complex exhibits significantly more favorable interaction energies compared to the SARS‐CoV/ACE2 complex, with the exception of desolvation energy. The positive desolvation energy observed in the MERS‐CoV/DPP4 complex is consistent with the estimates reported in reference [40]. This increase in desolvation energy may be attributed to strong hydration, likely resulting from the presence of highly solvated polar or charged residues at the protein–protein interface. Although this slightly reduces the favorability of the docking score, it is not sufficient to significantly hinder the formation of the MERS‐CoV/DPP4 complex, due to the dominance of other stabilizing interaction terms, as shown in Table 1.

The potential use of the DPP4 receptor by SARS‐CoV, in comparison with ACE2, can be inferred by analyzing their energy components as detailed in Table 1. Specifically, the SARS‐CoV/DPP4 complex shows more favorable van der Waals interactions but significantly less favorable electrostatic interactions compared to the SARS‐CoV/ACE2 complex. This imbalance is likely due to several repulsive interactions occurring at the binding interface of the SARS‐CoV/DPP4 complex, as detailed in Table S1. Although the SARS‐CoV/ACE2 complex exhibits more favorable interaction energies compared to the SARS‐CoV/DPP4 complex, the shape complementarity does not appear to play a predominant role in SARS‐CoV/ACE2, as it has less favorable van der Waals energy compared to SARS‐CoV/DPP4. In fact, data in Table 1 suggest that electrostatic interactions form a more favorable electrostatic complementarity [41, 42] in the binding region for SARS‐CoV/ACE2 compared to SARS‐CoV/DPP4.

The Table 1 also indicates that MERS‐CoV/DPP4 binding is highly driven by electrostatic complementarity, which is supported not only by the presence of salt bridges but also by charged residues at the interfacial surface. We may speculate that MERS‐CoV has evolved to exploit the charge‐mediated interactions provided by the DPP4 receptor.

### 3.2 SARS‐CoV/DPP4 Dissociation Dynamics

**FIGURE 4:**
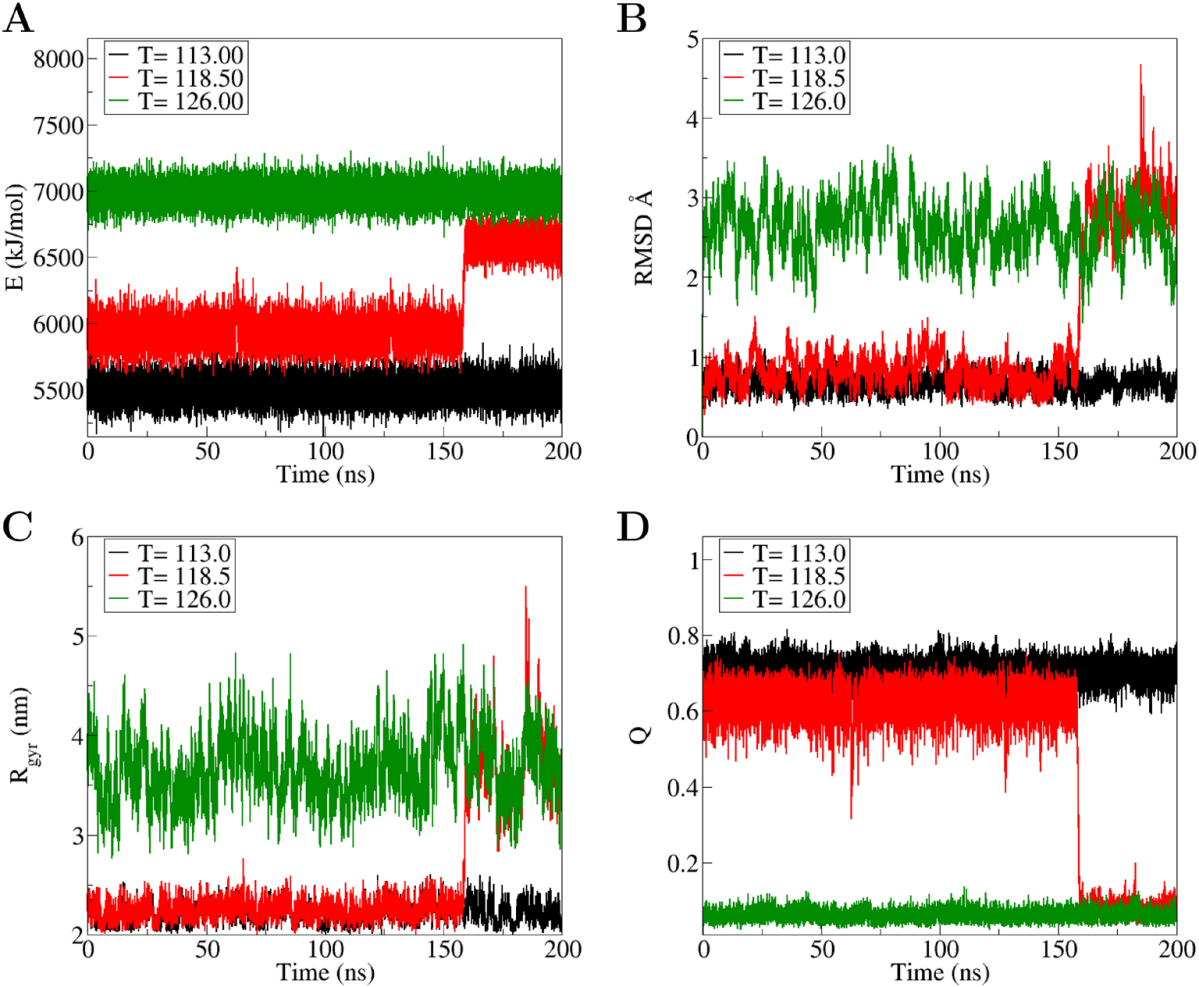
Time series from the MD simulations of the SARS‐CoV/DPP4 complex illustrating the bound, dissociation, and unbound states. (A) total energy E, (B) root‐mean square deviation (RMSD), (C) radius of gyration (*R*_gyr_), and (D) fraction of native intermolecular contacts (Q). The dissociation of the molecular complex from bound to unbound states occurs at a temperature of $T_{d}∼118.50$ (in reduced units), as indicated by the maximum in the thermal capacity $C_{V}$, shown in the inset of Figure 5. This dissociation is driven by the breaking of noncovalent interactions, resulting in the increasing of the total energy, radius of gyration, and RMSD. In contrast, the reaction coordinate Q decreases rapidly over time at $T_{d}$. The time series at temperatures below and above $T_{d}$ exhibit distinct behaviors, with the molecular complex remaining either in the bound state (at lower temperatures) or in the unbound states (at higher temperatures).

SBM modeling simulations were employed to investigate the dissociation dynamics of the SARS‐CoV/DPP4 complex. These simulations generated time series data for total energy, root‐mean‐square deviation, radius of gyration, and the fraction of native intermolecular contacts across 32 carefully chosen temperatures. Figure 4 presents time series for these quantities at three representative temperatures, highlighting their overall behavior. The results reveal a two‐state mechanism that governs the dissociation dynamics, characterized by a transition from an ordered to a disordered state.

This two‐state mechanism is evident from the abrupt changes observed in all the monitored quantities in Figure 4 for the time series generated at 118.5. The abrupt displacement observed around 160 ns at this temperature indicates the onset of protein–protein dissociation, leading to a new state characterized by higher energy, RMSD, *R*_gyr_, along with a lower fraction of native intermolecular contacts. This new state dominates the behavior of time series generated at higher temperatures, as exemplified by T=126. The time series at T=118.5 highlights the sharp nature of the transition and provides insights into the structural and energetic rearrangements underlying the dissociation process.

**FIGURE 5:**
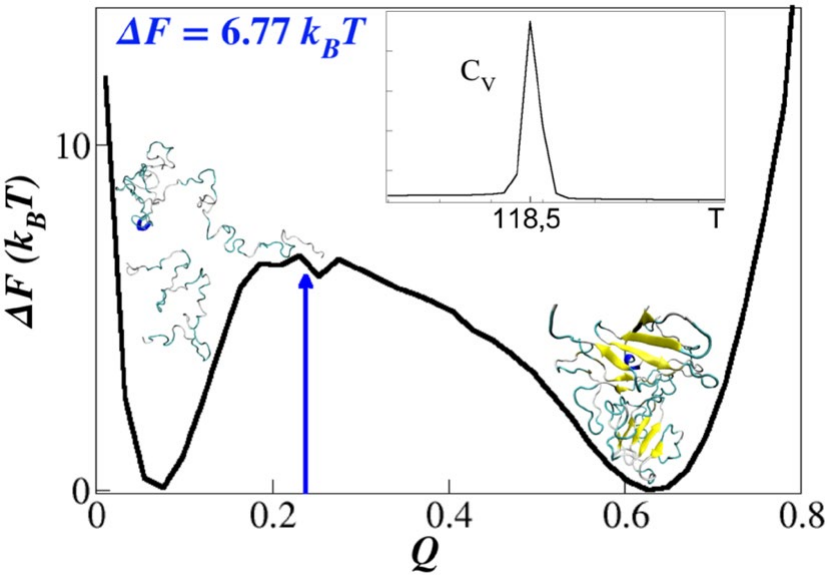
Free‐energy profile of dissociation as a function of the reaction coordinate Q, obtained from the SARS‐CoV/DPP4 simulations. The profile indicates that the dissociation dynamics follow a two‐state mechanism. The bound state corresponds to Q∼0.65, while the unbound state is observed at Q∼0.08. Inset: Heat capacity as a function of temperature.

The binding free‐energy profile $\Delta F\left(Q\right)$ of the SARS‐CoV/DPP4 complex was evaluated as a function of the fraction of native intermolecular contacts Q because it is known that this coordinate is a suitable measure for monitoring protein conformational dissociation. Figure 5 illustrates the thermodynamic free‐energy barrier that separates the bound and unbound states of the SARS‐CoV and DPP4 molecular structures. The inset graph of Figure 5 displays the thermal capacity $C_{V}$, which exhibits a maximum occurring at a temperature of $T_{d}=118.50$, in reduced units. The complex remains in thermal equilibrium at temperatures below $T_{d}$, preserving its native‐like structure. However, at temperatures above $T_{d}$, the complex undergoes a rapid transition into two independent, disordered conformations, corresponding to the spike protein and its receptor DPP4.

The complexation is characterized by $Q_{\text{bound}}∼0.65$, indicating the effective average fraction of native intermolecular contacts necessary to form the stable bound state. In contrast, the unbound state generated within our confined simulation box, exhibits a significantly lower fraction of native intermolecular contacts, $Q_{\text{unbound}}∼0.08$, reflecting its dissociated nature. Furthermore, the analysis of the free‐energy barrier, computed as $\Delta F=6.77\;k_{B}T$, suggests that the dissociation transition between the bound and unbound states involves a considerable energetic cost, implying that typical thermal fluctuations are insufficient to induce this state change under normal conditions.

### 3.3 SARS‐CoV/ACE2 Dissociation Dynamics

**FIGURE 6:**
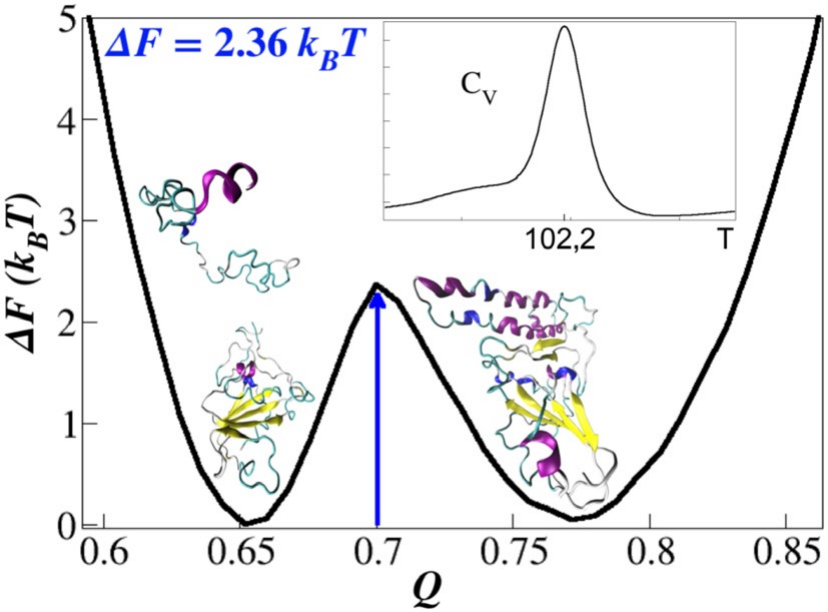
The free‐energy profile $\Delta F\left(Q\right)$ obtained from the SARS‐CoV/ACE2 simulations reveals that the dissociation dynamics follow a two‐state mechanism. The bound state corresponds to Q∼0.77, while the unbound state is observed at Q∼0.65. Inset: Thermal capacity $C_{V}$ as a function of temperature.

The SBM modeling simulations were also employed to study the dissociation dynamics of SARS‐CoV/ACE2 complex for comparative purposes, using the same force field. The time series for the total energy, RMSD, $R_{\mathrm{gyr}}$, and Q, are presented in Figure S3. The free‐energy landscape as a function of the fraction of native intermolecular contacts is shown in Figure 6. This figure highlights the transition between the ordered and disordered states of the SARS‐CoV/ACE2 complex, characterized by a free‐energy barrier of $\Delta F=2.36\;k_{B}T$. As the temperature increases, the molecular chains involved in the complexation undergo a dissociation transition at a temperature of $T_{d}=102.20$, as determined by the maximum of the thermal capacity $C_{V}$ (inset of Figure 6). The native‐like structures of this complex remain in thermal equilibrium at temperatures below $T_{d}$, with $Q_{\text{bound}}∼0.77$. In contrast, above $T_{d}$, the system transitions rapidly to two independent disordered conformations, characterized by $Q_{\text{unbound}}∼0.65$, which correspond to the spike and receptor proteins.

### 3.4 MERS‐CoV/DPP4 Dissociation Dynamics

The SBM modeling simulations of the MERS‐CoV/DPP4 complex produced time series data for the total energy, RMSD, *R*_gyr_, and Q, as shown in Figure S4.

**FIGURE 7:**
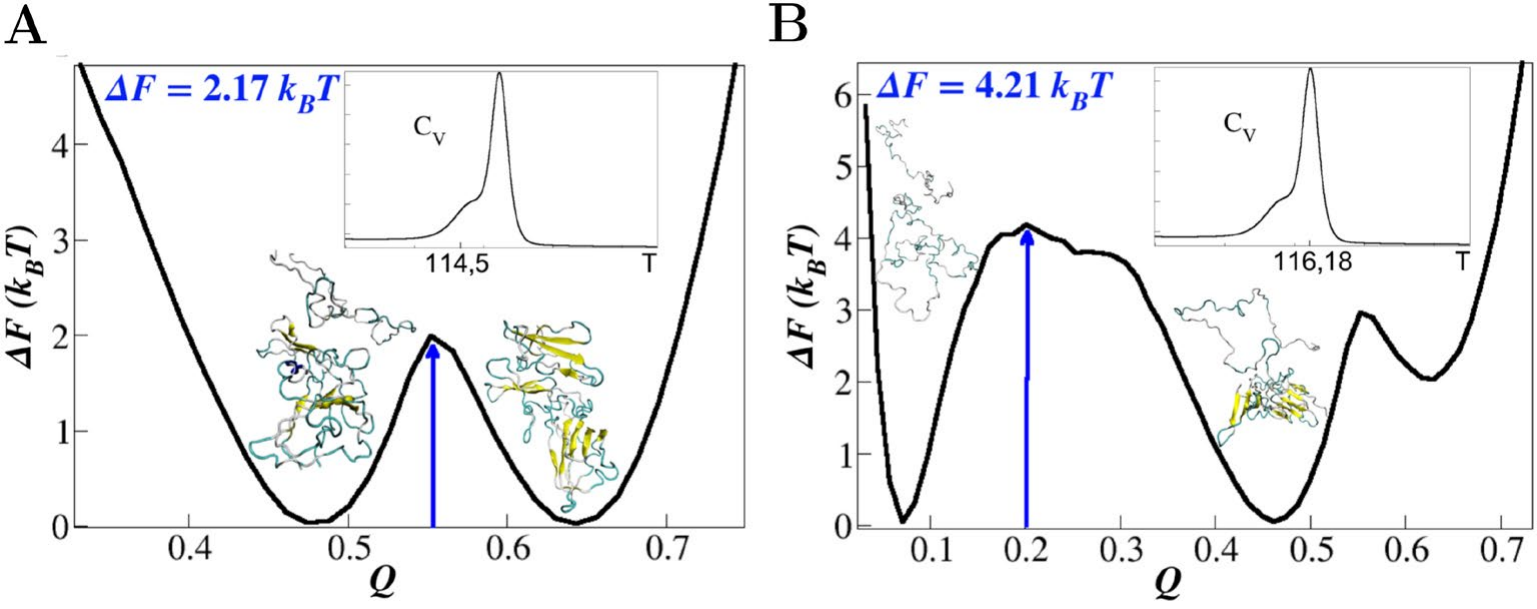
Free‐energy profile $\Delta F\left(Q\right)$ for the MERS‐CoV/DPP4 complex, highlighting a two‐step dissociation process. (A) The initial dissociation transition occurs at $T_{d}^{A}=114.5$, with the partially unbound state characterized by $Q_{\text{unbound}}^{A}∼0.47$. This step involves overcoming a relatively small free‐energy barrier, lower than that observed for the SARS‐CoV/ACE2 complex. (B) The complete dissociation, accompanied by substantial structural changes, occurs at a slightly higher temperature, $T_{d}^{B}∼116.5$. This second transition involves overcoming a higher free‐energy barrier.

The thermal capacity $C_{V}$, shown in the insets of Figure 7A,B, reveals a two‐step process for the dissociation transition to the unbound states as the temperature increases. These two transitions result in the free‐energy profiles $\Delta F\left(Q\right)$ depicted in Figure 7A,B.

The first transition corresponds to a small peak in $C_{V}$ (Figure 7A), which can be interpreted as an initial dissociation event preceding the complete unfolding of the proteins. This transition occurs at a temperature of $T_{d}^{A}=114.50$, with a relatively low free‐energy barrier of $\Delta F^{A}=2.17\;k_{B}T$. The bound state of the complex is characterized by $Q_{\text{bound}}^{A}∼0.64$, while the partially unbound state retains a significant portion of its network of native contacts, with $Q_{\text{unbound}}^{A}∼0.47$.

Figure 7B illustrates the free‐energy landscape related to the complete dissociation of that complex, characterized by $Q_{\text{unbound}}^{B}∼0.07$ and significant unfolding of the proteins. This second transition is associated with the maximum in $C_{V}$ occurring at a slightly higher temperature of $T_{d}^{B}=116.18$, compared to $T_{d}^{A}$. The final dissociation needs to overcome a slightly higher free‐energy barrier of $\Delta F^{B}=4.21\;k_{B}T$, to promote a substantial structural change in the proteins.

## 4 Discussion

The energetic components presented in Table 1 for the SARS‐CoV/DPP4, SARS‐CoV/ACE2, and MERS‐CoV/DPP4 complexes provide insights into the mechanisms of protein‐receptor binding. These results reveal distinct structural binding mechanisms for each complex. The binding energies are characterized by short‐range van der Waals and electrostatic interactions. The short‐range interactions are dominated by hydrophobic residues located at the protein–protein interface, where side chain rearrangements can lead to the shape complementarity already established for the SARS‐CoV/ACE2 complex [26].

Our analysis indicates that electrostatic complementarity plays a significant role in the interaction between MERS‐CoV and DPP4, as evidenced by the presence of salt bridges and charged residues at the binding interface. However, this binding mechanism appears to be less effective for SARS‐CoV binding to DPP4. In fact, electrostatic complementarity is more favorable between SARS‐CoV and ACE2 than between SARS‐CoV and DPP4.

Interestingly, Grassmann et al. [42] noted an inverse relationship between electrostatic complementarity and the stability of protein–protein complexes. Our data corroborate this observation: higher electrostatic complementarity correlates with lower stability, as measured by the free‐energy barrier of dissociation. This counterintuitive result may be explained by considering the balance of forces contributing to the overall stability of the complexes. While electrostatic interactions can strengthen the protein–protein interaction, they may also lead to frustration due to competing interactions. This could make the complex more sensitive to physicochemical conditions such as changes in pH, ionic strength, or temperature, which could significantly reduce stability and make the complex easier to dissociate.

The stability of those complexes was evaluated using MD simulations. The SARS‐CoV/DPP4 complex exhibited the highest free‐energy barrier, $\Delta F=6.77\;k_{B}T$, followed by SARS‐CoV/ACE2 at $\Delta F=2.36\;k_{B}T$, and MERS‐CoV/DPP4, $\Delta F=2.17\;k_{B}T$. These results suggest that the lower stability of the MERS‐CoV/DPP4 complex makes it the most likely to form, and also, the complex least resistant to dissociation.

The SARS‐CoV/ACE2 complex showed the highest $Q_{\text{bound}}$, indicative of well‐organized interfacial side chains that facilitate H‐bond interactions. However, this complex exhibited the lowest dissociation temperature and a relatively small free‐energy barrier, making it more prone to dissociation compared to the other complexes analyzed. In contrast, the SARS‐CoV/DPP4 complex exhibited the highest free‐energy barrier, although $Q_{\text{bound}}$ is comparable to the MERS‐CoV/DPP4 complex. This suggests that, while the formation of SARS‐CoV/DPP4 is less likely, once formed, it exhibits significant stability. As a result, the dissociation is delayed until substantial conformational changes or unfolding occur at slightly higher temperatures compared to MERS‐CoV/DPP4 complex.

In conclusion, our findings suggest that DPP4 may function as a coreceptor for SARS‐CoV. Nonetheless, the SARS‐CoV/DPP4 complex is less prone to dissociation, though its energetic profile does not make such dissociation prohibitive. In fact, we cannot dismiss the possibility that specific genetic variations in DPP4, particularly, those affecting charged residues, could enhance the electrostatic interaction between SARS‐CoV and the DPP4 receptor. This is because low pH conditions can enhance electrostatic protein–protein interactions by protonating acidic residues, reducing repulsion, and optimizing attractive interactions between oppositely charged residues. Experimental studies have shown that certain DPP4 polymorphisms, which involve changes in charged residues, can diminish the binding efficiency of MERS‐CoV to DPP4 [43]. It would be interesting to investigate whether these DPP4 variants could reduce the dissociation energy in the SARS‐CoV/DPP4 complex. Moreover, elevated levels of carbon dioxide in the blood (hypercapnia), often resulting from severe viral respiratory infections, can lower blood pH and contribute to respiratory acidosis [44, 45].

This nuanced understanding of protein‐receptor interactions provides valuable insights into the functional roles of DPP4 and ACE2 in mediating viral entry. Such knowledge can support the development of therapeutic strategies targeting these complexes. Specifically, peptides or small molecules engineered to mimic the interface residues of DPP4, or its rationally designed variants, may be employed to reduce the association constant ($K_{a}$), thereby destabilizing the SARS‐CoV/DPP4 and MERS‐CoV/DPP4 complexes and inhibiting productive viral engagement.

## Author Contributions

**Patrícia Pereira Duzi Carvalho:** methodology, software, data curation, validation, writing – original draft, conceptualization. **Nelson Augusto Alves:** conceptualization, methodology, project administration, writing – original draft, writing – review and editing, software.

## Conflicts of Interest

The authors declare no conflicts of interest.

## Supporting information


**Data S1.** Supporting Information.

## Data Availability

The data that supports the findings of this study are available in the supplementary material of this article.
