# Supplementary material for: Role of the DPP4 Receptor in SARS‐CoV Entry: Insights From Docking and Molecular Dynamics Simulations
Source: Proteins. 2025 Jul 2;93(12):2091–100. doi: 10.1002/prot.70011 (PMC12594196; doi:10.1002/prot.70011)
Supplement: Supplementary file 1 — Data S1. Supporting Information. [file PROT-93-2091-s001.pdf]

Role of the DPP4 receptor in SARS-CoV entry: Insights from docking and molecular dynamics simulations

1 Supporting Information

1.1 Docking process of SARS-CoV and DPP4

The SARS-CoV/DPP4 docking was generated using the full crystallographic structures of the SARS-CoV RBD, specified by residues 323-502 (PDB ID:2AJF), and the DPP4 conformation, specified by residues 39-766 (PDB ID:1NU6), as templates.

The docking process comprised three main steps: rigid-body docking, semi-flexible refinement, and final optimization in explicit solvent. Initially, approximately 1,000 molecular complex structures were generated, and the top 200 were selected for further refinement. This refinement involved semi-flexible simulated annealing in torsion angles, followed by a short restrained molecular dynamics simulation in an explicit solvent. Clustering was then performed with a 7.5 Å cut-off and a minimum cluster size of 4 structures, resulting in the grouping of 200 structures into 10 clusters. These clusters were analysed and ranked based on the HADDOCK-score, which represents the average interaction energies (sum of $E_{vdw}, E_{elec}, E_{desolv}, E_{air}$) within each cluster.

The docked structures underwent additional evaluation based on the analysis of the RMSD among the 40 best docked poses. The strucuture with PDB ID:2AJF was used as an experimental reference to overlay the docking result, yielding an RMSD of 0.426 Å for the complex. Our final conformation is shown in Figure S1B.

1.2 Structure validation of modeled proteins

The quality of the constructed models was assessed in terms of stereochemical properties using the structural evaluation tool provided by the MolProbity web server (`http://molprobity.biochem.duke.edu/`) [1]. The analysis indicated that 91.3% (825/904) of all residues were located in the favored regions (98%), and 98.9% (894/904) of all residues fell within the allowed regions (greater than 99.8%) (see Figures S1A and S2).

Additionally, the RMSD between our model and the target experimental structure 2AJF was measured at 0.426 Å, signifying that the backbone geometry of the model was optimal.

1.3 rCSU - Description for Contact Maps

This study employs CSU-related algorithms (`http://info.ifpan.edu.pl/~rcsu/rcsu/index.html`) to generate comprehensive contact maps for proteins [2]. Two primary contact map types are produced: the original contact map (oCSU) and an improved variant (rCSU). The rCSU map specifically differentiates between favorable (proper) and unfavorable (destabilizing) atomic contacts, declaring residues in contact only if the former outnumber the latter, and also identifies ionic bridges.

A **B**

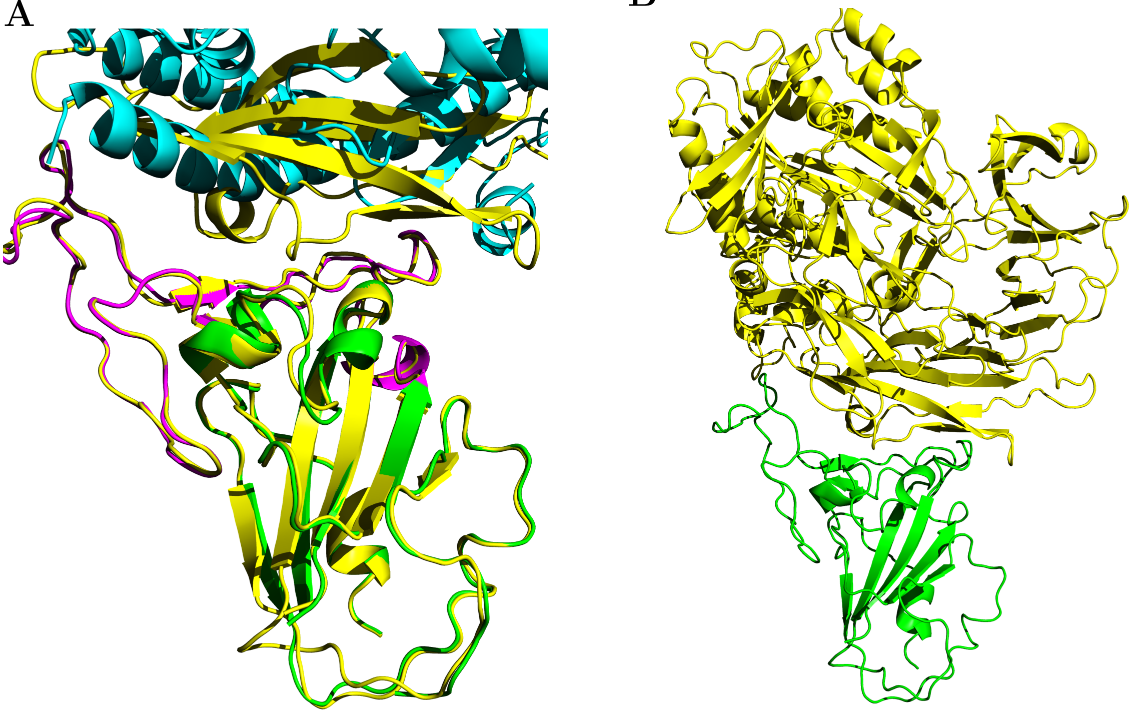

Figure S1: Figure (A) exhibits the overlay of the SARS-CoV crystallographic structure (RBD in green and the RBM in magenta) over the SARS-CoV/DPP4 molecular complex (in yellow) predicted by HADDOCK modeling. The ACE2 crystallographic structure is shown in cyan. (B) The complete molecular docking prediction between SARS-CoV (in green) and the DPP4 receptor of MERS-CoV (in yellow).

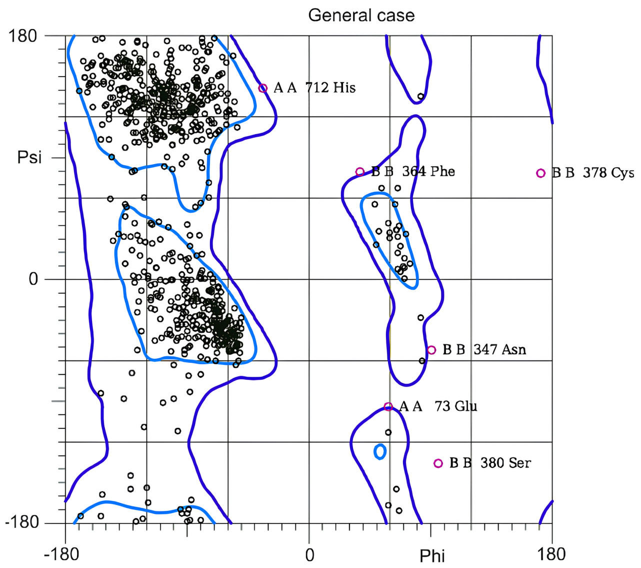

Figure S2: Statistical validation of SARS-CoV/DPP4 modeling through the Ramachandran MolProbity plot.

References

[1] Williams CJ, Headd JJ, Moriarty NW, Prisant MG, Videau LL, Deis LN. MolProbity: More and better reference data for improved all-atom structure validation. *Protein Sci.* 2018; 27(1):293–315. DOI: 10.1002/pro.3330

[2] Wolek K, Gómez-Sicilia À, Cieplak M. Determination of contact maps in proteins: A combination of structural and chemical approaches. *J Chem Phys.* 2015; 143(24):243105. DOI: 10.1063/1.4929599

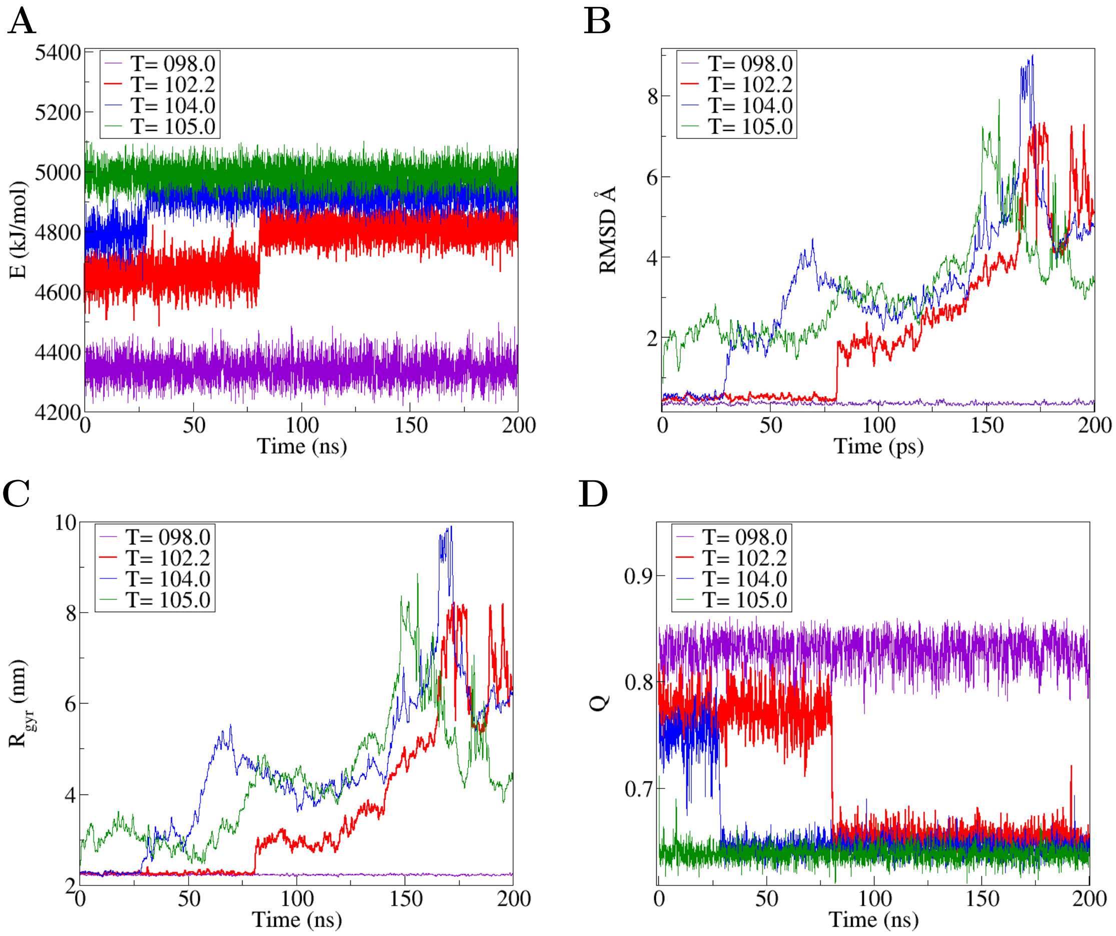

Figure S3: Time series from the SARS-CoV/ACE2 simulations. (A) energy, (B) root-mean-square deviation RMSD, (C) radius of gyration R_{gyr}, and (D) fraction of native intermolecular contacts Q. The dissociation from bound to unbound states occurs at $T_d = 102.2$. The temperature values are expressed in reduced units.

Contacts	Distance (Å)	specific contacts	Contacts	Distance (Å)	specific contacts
R426/R336	5.77, 5.53	Nc, Dc	D480/A291	3.78, 3.22, 2.98	Hb, Nc, Dc
S432/K267	5.29	Hb	D480/L294	3.77	Dc
T433/K267	4.49	Hb	D480/I295	3.79, 4.24	Dc, Nc
Y436/L294	3.92, 3.49	Dc, Ph	Y481/A291	4.20, 5.01, 4.43, 5.37	Nc, Hb, Dc, Ph
Y440/A291	3.45, 4.49, 3.44	Ph, Hb, Dc	Y481/L294	3.41	Dc
Y440/S292	4.82	Hb	G482/T288	4.51, 4.20	Nc, Hb
Y440/V341	4.74, 3.83	Ph, Dc,	G482/L294	3.27, 3.62	Nc, Dc
Y440/Q344	5.0	Hb	Y484/K267	3.42, 3.37, 2.94	Dc, Nc, Hb
Y442/S292	4.01, 4.03	Dc, Nc	Y484/Q286	3.19	Dc
Y442/R317	2.80	Hb	Y484/T288	4.27, 3.47	Dc, Ph
Y442/Y322	3.96, 3.18	Ar, Dc	T485/R336	5.17	Hb
Y442/I346	5.96, 5.20	Dc, Ph	T486/F269	6.01	Nc
L443/Y322	4.05, 3.40	Ph, Dc	T486/S284	4.69, 5.74	Dc, Nc
L443/I346	5.03	Ph	T486/I285	4.69	Nc
L443/M348	6.09, 6.43	Nc, Ph	T486/Q286	3.39, 2.65, 3.31	Nc, Hb, Dc
F460/M348	4.12, 3.93	Nc, Ph	T486/R336	4.83, 3.96	Nc, Hb
D463/T350	4.60, 4.11, 4.09	Nc, Hb, Dc	T487/I285	4.89	Nc
K465/T350	5.67	Dc	T487/Q286	3.56, 3.37, 3.44	Hb, Nc, Dc
P469/E677	5.53	Dc	T487/I287	4.66	Nc
P470/I319	4.10, 3.63	Ph, Dc	T487/T288	3.28	Dc
P470/L673	5.46	Ph	T487/R336	3.92, 2.83	Nc, Hb
P470/T675	5.50	Dc	G488/I285	4.53, 4.49	Dc, Nc
P470/E677	3.74,4.28	Ph, Dc	G488/Q286	3.74	Hb
P470/D678	5.09, 5.09, 4.87	Nc, Dc, Ph	G488/R336	3.61	Nc
A471/I319	5.45	Dc	G488/W337	3.85, 3.32	Hb, Nc
A471/N321	4.55	Hb	I489/D331	3.86, 3.58, 4.07	Nc, Dc, Ph
A471/R596	6.25	Nc	I489/S334	3.59, 3.61	Dc, Nc
A471/D678	6.09	Nc	I489/R336	3.40, 3.76, 4.91	Dc, Ph, Nc
L472/I319	4.04	Ph	I489/N338	3.41, 4.11	Dc, Nc
L472/N321	3.36, 3.61, 3.85	Nc, Dc, Ph	G490/N338	2.78, 3.36	Hb, Nc
L472/S349	4.79	Dc	Y491/Q286	5.43	Dc
L472/T350	4.68	Dc	Y491/I287	5.45, 5.16	Nc, Ph
L472/G352	4.48	Dc	Y491/T288	3.39, 4.00, 3.07	Dc, Nc, Hb
L472/N595	3.93	Dc	Y491/A289	4.97, 3.84	Dc, Hb
L472/R596	4.55, 5.29	Nc, Dc	Y491/P290	3.85, 3.86	Nc, Dc
N473/N321	3.04	Dc	Y491/A291	4.89	Hb
N473/M348	5.81	Dc	Y491/D326	3.92	Hb
N473/S349	4.45, 3.67, 4.68	Nc, Dc, Hb	Y491/W337	4.73	Dc
N473/T350	4.96, 3.27, 3.37	Ph, Nc, Dc	Y491/N338	4.59, 4.54, 4.61	Dc, Hb, Nc
C474/R317	4.50	Hb	Y491/C339	3.83, 3.53, 3.59, 4.96	Dc, Nc, Ph, Hb
C474/I319	5.96	Dc	Y491/V341	5.44	Dc
C474/N321	4.54, 5.37	Dc, Hb	Y491/Q344	5.31	Hb
Y475/R317	3.95, 3.36	Nc, Dc	Q492/R336	5.81	Hb
Y475/N321	3.48, 3.13	Dc, Hb			
Y475/Y322	3.55, 3.52	Ph, Ar			
Y475/M348	3.51, 3.95	Nc, Dc			
Y475/S349	4.83	Hb			
W476/I295	4.48	Ph			
W476/R317	4.66	Dc			
L478/I295	4.46, 3.51, 5.46	Nc, Dc, Ph			
N479/A291	4.37, 3.68, 3.33	Nc, Ph, Dc			
N479/S292	2.98, 2.92, 3.00	Nc, Hb, Dc			
N479/I295	4.81, 5.09	Nc, Dc			
N479/R317	4.61	Hb			

Table S1: SARS-CoV/DPP4 interfacial contacts. Contacts and distances are measured between atom pairs at interchain residues. The type of interaction depends on the participating atoms in the interaction and the distance between the pairs of atomic contacts. Type of interaction: hydrogen bond (Hb), hydrophobic (Ph), aromatic (Ar), salt bridge (Ib), repulsive (Dc), denotes other insignificant contacts (Nc).

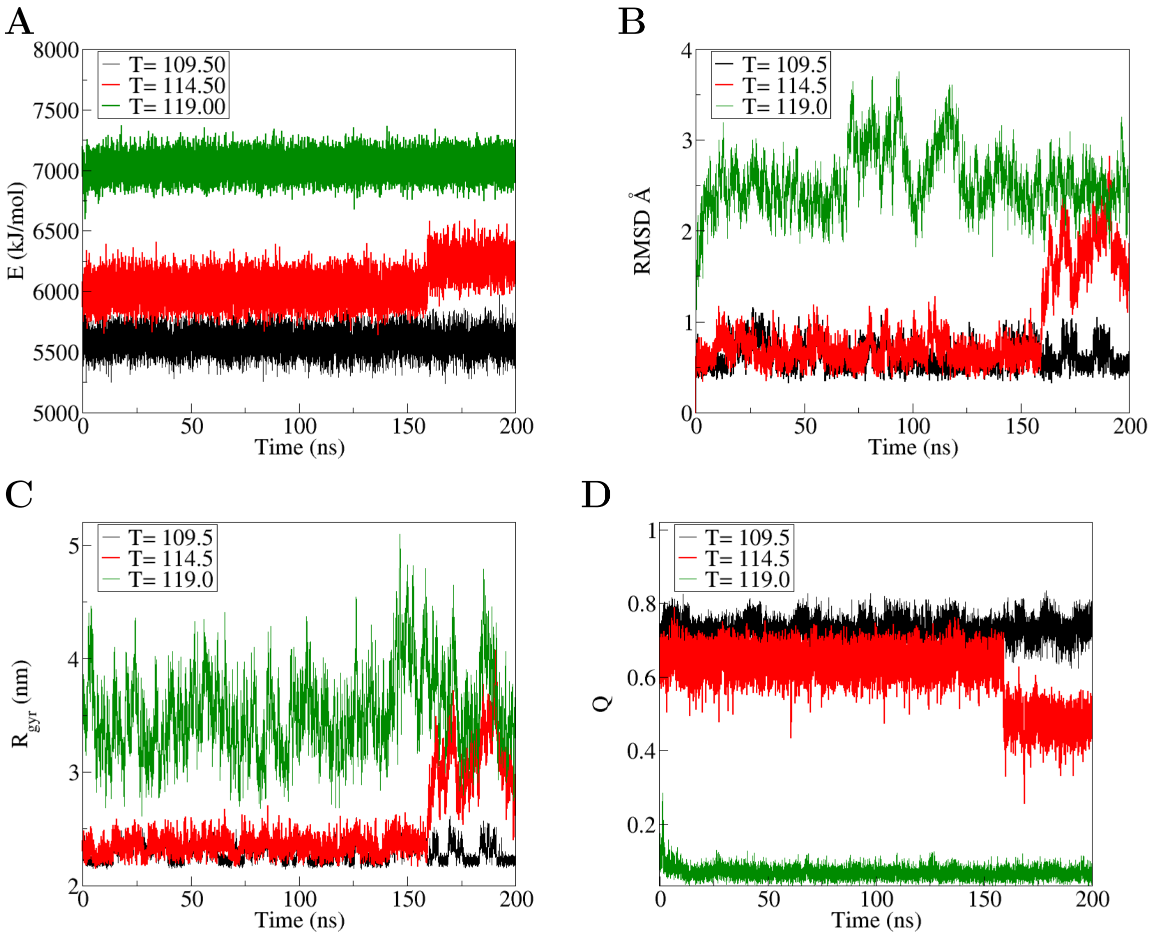

Figure S4: Time series from the MERS-CoV/DPP4 simulations. (A) total energy, (B) root-mean-square-deviation RMSD, (C) radius of gyration R_{gyr}, and (D) fraction of native intermolecular contacts Q. The dissociation from bound to unbound states occurs at $T_d \sim 114.5$.
